# Polymeric DNA Hydrogels and Their Applications in Drug Delivery for Cancer Therapy

**DOI:** 10.3390/gels9030239

**Published:** 2023-03-18

**Authors:** Jing Li, Wenzhe Song, Feng Li

**Affiliations:** 1 Frontiers Science Center for Synthetic Biology, Institute of Biomolecular and Biomedical Engineering, Key Laboratory of Systems Bioengineering (MOE), School of Chemical Engineering and Technology, Tianjin University, Tianjin 300350, China; 2 State Key Laboratory of Chemical Resource Engineering, Beijing Advanced Innovation Center for Soft Matter Science and Engineering, College of Chemistry, Beijing University of Chemical Technology, Beijing 100029, China

**Keywords:** DNA hydrogel, DNA nanotechnology, drug delivery, cancer therapy

## Abstract

The biomolecule deoxyribonucleic acid (DNA), which acts as the carrier of genetic information, is also regarded as a block copolymer for the construction of biomaterials. DNA hydrogels, composed of three-dimensional networks of DNA chains, have received considerable attention as a promising biomaterial due to their good biocompatibility and biodegradability. DNA hydrogels with specific functions can be prepared via assembly of various functional sequences containing DNA modules. In recent years, DNA hydrogels have been widely used for drug delivery, particularly in cancer therapy. Benefiting from the sequence programmability and molecular recognition ability of DNA molecules, DNA hydrogels prepared using functional DNA modules can achieve efficient loading of anti-cancer drugs and integration of specific DNA sequences with cancer therapeutic effects, thus achieving targeted drug delivery and controlled drug release, which are conducive to cancer therapy. In this review, we summarized the assembly strategies for the preparation of DNA hydrogels on the basis of branched DNA modules, hybrid chain reaction (HCR)-synthesized DNA networks and rolling circle amplification (RCA)-produced DNA chains, respectively. The application of DNA hydrogels as drug delivery carriers in cancer therapy has been discussed. Finally, the future development directions of DNA hydrogels in cancer therapy are prospected.

## 1. Introduction

Hydrogels are three-dimensional physical or chemical polymer networks. Hydrogels are composed of hydrophilic groups and are capable of absorbing and swelling in water while not soluble in water, which endows hydrogels with the properties of biocompatibility, viscoelasticity, and certain mechanical strength [1,2,3]. Due to their excellent properties, hydrogels have been extensively used in drug delivery and tissue engineering [4,5,6]. Hydrogels can be prepared from various types of polymers, including both natural and synthetic organic polymers. Natural hydrogels, which mainly consist of polymers, have been extensively studied in the past decades, for example, collagen is one of the most representative natural hydrogel materials, and has been investigated for use in the reconstruction of skin and blood vessels [7,8]. In addition, hydrogels originated from natural polymers, such as fibrin and alginate, have been developed as biocompatible materials for wound healing and drug delivery in tissue engineering. Nevertheless, most natural polymer hydrogels lack elasticity and are easy to degrade in vivo, which would restrict their applications as drug carriers [9,10]. Synthetic polymer-based hydrogels were then developed to enhance the stability and improve the property of hydrogels, and crosslinking agents were the decisive substances to the permeability and hydrophilicity of the hydrogels [11,12,13,14]. Poly(2-hydroxyethyl methacrylate) (HEMA) was the most studied crosslinking agent, as early as 1960, WiChterle and Lim synthesized cross-linked HEMA hydrogels [15]. Later in 1995, Em Ende and Peppas prepared ionizable pH-sensitive hydrogels using acrylic acid (AA) and HEMA as original materials, with ethylene glycol dimethacrylate as a crosslinking agent. They investigated the diffusion of solutes, such as drugs and proteins in the porous network of hydrogel, as well as the changes in the hydrogel formation under environmental stimuli [16]. Apart from synthetic polymers introduced above, poly (vinyl alcohol) (PVA) and poly(ethylene glycol) (PEG) were used to prepare hydrogels as well [17]. Another widely used and representative organic polymer for the preparation of hydrogel was poly (N-isopropylacrylamide) (PNIPAM), whose lower critical solution temperature (LCST) was 32 °C and could finish phase transition from liquid under LCST to solid hydrogel state above the LCST [18,19]. The use of PNIPAM as an original material for hydrogel propelled the development of temperature-responsive hydrogels and made the stimuli-responsive hydrogels a research hotspot [20]. We recently reported the fabrication of a bioinspired hydrogel with unique mechanical responsiveness, the hydrogel was developed via the copolymerization of N-hydroxyethylacrylamide with a dynamic coordination system composed of telluroether (Te) monomer and platinum (Pt) ion. The established responsive hydrogel exhibited switchable and tunable porous structures and mechanical properties, which enriched the development of mechanically responsive and deformable materials [21]. In addition to chemically cross-linked hydrogels, supramolecular hydrogels formed via physical interactions between molecules were also developed rapidly. The assembly of supramolecular hydrogels is based on multiple weak interactions, which endows the hydrogels with reversible sol-gel transition ability and excellent stimuli-responsiveness property [22]. Peptides- and proteins-based hydrogels are a typical class of supramolecular hydrogels. Tang et al. reported a nanofiber-like hydrogel formed by pentapeptides. The hydrogel showed adjustable mechanical properties through changing the sequence of amino acid, pH value of solution, and peptide concentration, demonstrating the promising applications in cell delivery and tissue engineering [23]. Besides, polysaccharides, nucleic acids, and synthetic polymers can also be bound together to form supramolecular hydrogels via hydrogen bonding, hydrophobic interactions, electrostatic interactions, etc. [24,25,26].

With the development of DNA (Deoxyribonucleic acid) nanotechnology, DNA has been used as a promising building block for the preparation of abundant two/three dimensional biomaterials, among which, DNA hydrogels have been established and widely studied [27]. DNA hydrogels are DNA-based soft materials composed of polymeric networks on the basis of cross-linked DNA chains, which belongs to the category of typical supramolecular hydrogels [28]. The first DNA hydrogel was designed and prepared by Nagahara and Matsuda in 1996 [29]. According to the components, DNA hydrogels can be classified into pure DNA hydrogel and hybrid DNA hydrogel [30]. In addition, DNA hydrogels also can be classified into macron sized bulk hydrogels and sub-micron sized nanogels according to their size scale. The precise base pairing principle of DNA molecules allows for precise sequence programmability, structural controllability and exceptional molecular recognition ability. These biological properties make DNA an attractive biomaterial for the “customization” of DNA-based polymers [31]. DNA has been widely used in biomedical fields such as disease diagnosis, protein engineering, drug and gene delivery, disease treatment, cell engineering, etc. [32]. DNA hydrogels keep high water-content and exhibit excellent biocompatibility, making them suitable biomaterials. Additionally, DNA modules within the hydrogel possess unique recognition abilities, enabling them to directly target biomarkers on cell surface. These properties of DNA hydrogels provide significant advantages in the bioengineering field.

It is imperative that a precise and controllable drug delivery system play an extremely significant role in cancer treatment [33]. Currently, plenty of drug delivery systems have been developed for the treatment of cancer. From the first oral controlled release formulation Spansule developed by Smith et al. in 1950 to today’s lipid nanoparticles (LNP) for delivery of RNA interference drugs [34], remarkable research achievements have been acquired in the drug delivery field, and the drug sustained release time reached 24 weeks from the initial 24 h, which showed the great improvement of drug delivery systems [17]. The early drug delivery systems focused on developing controlled release formulations. In 1989, the U.S. Food and Drug Administration (FDA) approved the first long-acting injectable formulation Lupron Depot, which was based on poly(lactide-co-glycolide) (PLGA). PLGA-based formulations exhibited a long history of safety and were widely used over the past years [35]. However, the absence of characterization of PLGA limited the development of PLGA-derived polymers as drug carriers. Later, PEGylated liposomes were developed to deliver anti-cancer drugs for their lower immunogenic responses. Doxil was the first approved PEGylated liposomal formulation, which increased uptake of tumor and decreased drug toxicity of chemotherapeutic doxorubicin (DOX) [36,37]. Nevertheless, the delivery systems mentioned above had a drawback in common: the process of drug delivery was difficult to control, and the anti-cancer drugs would damage normal cells in vivo as well, which would simultaneously diminish the therapy efficiency. Thus, the problem was controlled drug encapsulation and release. In recent years, the development of nanotechnology has promoted the progress of drug delivery systems, which were termed as nanomedicine [1]. As commonly used carriers for drug delivery, nanoparticles such as polymersomes and micelles possess the ability to encapsulate a variety of drugs with controllability and the ability to be functionalized, thereby presenting ideal application prospects in cancer therapy [38,39]. However, these drug delivery carriers mainly consist of highly toxic organic polymers, which are difficult to degrade in vivo [40]. Therefore, it is crucial to develop biocompatible and biodegradable materials for drug delivery. With excellent biocompatibility and well mechanical properties, DNA hydrogels have been explored as drug delivery systems which can encapsulate and deliver a wide range of therapeutics, including small molecule drugs such as DOX and nucleotide drugs such as microRNA (miRNA), as well as biomacromolecules such as proteins, peptides, and stem cells, to the target sites in vivo. Moreover, as a kind of biomaterial derived from organisms, DNA could be degraded by nuclease over time [41,42,43]. Besides, DNA hydrogels have good biodegradability and thus present minimal toxic side effects to organisms. All these mentioned properties make DNA hydrogel an outstanding candidate for drug delivery. Previous studies have reviewed the applications of DNA hydrogels in the biomedical field, including molecular diagnostics, biosensing, cell culture, cancer therapy, etc. [28,44]. However, to the best of our knowledge, DNA hydrogel-based drug delivery system for cancer therapy has not been systematically reviewed.

In this review, we focus on the hydrogels which are formed by branched DNA modules, hybrid chain reaction (HCR)-synthesized DNA networks and rolling circle amplification (RCA)-produced DNA chains, respectively, and their applications in drug delivery for cancer therapy. We discuss and summarize their use in various modalities, including chemotherapy, gene therapy, immunotherapy, photo dynamic/thermal therapy, and cooperative therapy. The property of the three construction strategies of DNA hydrogels were summarized (Table 1). The representative works are summarized to show how to design DNA sequences to obtain different DNA hydrogels with appealing properties for applications in drug delivery and cancer therapy. In addition, the advantages and the limitations of DNA hydrogels are discussed. Finally, we thoroughly discussed the potential applications and future developments of DNA hydrogels in drug delivery for cancer therapy.

## 2. The Preparation and Drug Delivery Applications of DNA Hydrogels

### 2.1. Branched DNA Formed Hydrogel

#### 2.1.1. Branched DNA

Branched DNA is a synthetic structure with at least three DNA strands extending from each branched point. Distinct from linear DNA and circular DNA, branched DNA has a more complex topological structure, which can provide more flexible molecular component primitives for the constructing DNA functional materials [59,60]. With rational molecular design, branched DNA monomers could form cross-linked hydrogels.

**Figure 1:**
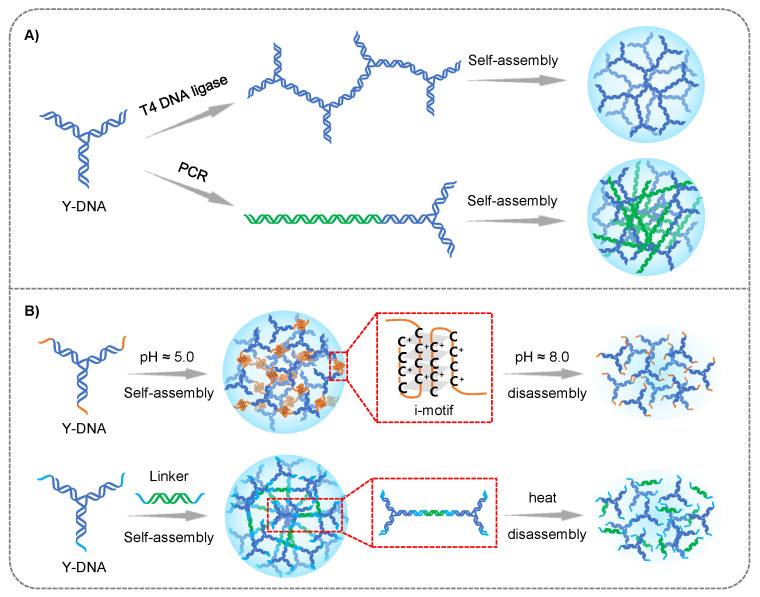
The preparation strategies of branched DNA hydrogels: (**A**) enzyme-mediated assembly. (**B**) non-enzyme-mediated assembly.

The construction strategies of DNA hydrogel from branched DNA can be divided into enzyme- and non-enzyme mediated assembly (Figure 1). In 2006, Luo et al. for the first time constructed hydrogels via efficient ligase-mediated assembly of branched DNA [61]. They designed three types of branched DNA monomers with palindromic complementary sticky ends to form DNA hydrogels through the T4 DNA ligase catalyzed DNA ligation. In 2013, Luo and colleagues developed another strategy of enzyme-catalyzed DNA hydrogel. They used thermostable branched DNA strands as modular primers for polymerase chain reactions (PCR). During the PCR, these primers were extended and connected to form a network structure [62].

In 2009, Liu group reported a pH-triggered DNA hydrogels, which was constructed by the formation of intermolecular i-motif structures without enzyme catalysis [63]. The i-motif was folded in acidic environment, so the sol-gel transformation could be accomplished by controlling the pH value of the solution. Afterwards, they created a pure DNA hydrogel assembled from a Y-scaffold and a linker DNA. The responsive temperature of the DNA hydrogel could be adjusted by tailoring the sticky ends of the building blocks, and the restriction sites could be inserted into the linker sequence to achieve the enzyme-mediated control of sol-gel transition [64]. In 2016, Nishida et al. reported a hydrogel assembled from Takumi-shaped DNA. The assembly process did not need enzymes, but instead of the complementary base pairing at the 5′ and 3′ ends of the Takumi [65]. Nevertheless, the formation of branched DNA-based hydrogel requires high DNA concentration due to the mono-DNA modules, which increases the preparation cost.

#### 2.1.2. Application in Cancer Therapy

Branched DNA hydrogels showed a great potential in drug delivery and tumor therapy due to their programmability, controllability, size-tunability, multivalency, and excellent molecular recognition ability [32]. The sequences of branched DNA strands can be designed to link with different functional elements, such as functional nucleic acids and proteins, which endowed branched DNA hydrogels with outstanding targeting and drug release capabilities.

**Figure 2:**
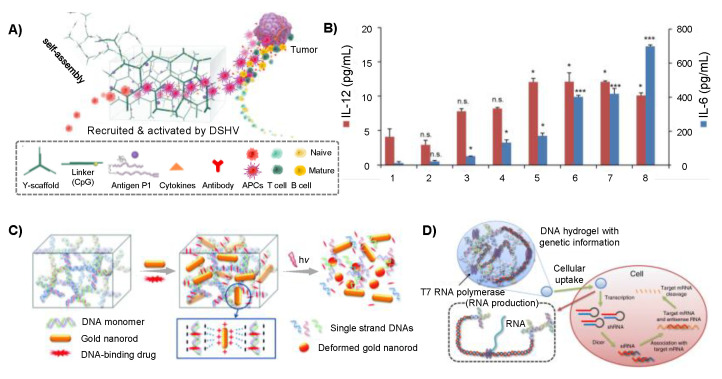
Branched DNA formed hydrogels for cancer therapy: (**A**) The recruitment and activation of APCs by the DNA supramolecular hydrogel vaccine (DSHV) system. (**B**) Different samples stimulated APCs to produce IL-6 and IL-12. Reproduced with permission. * *p* < 0.05, *** *p* < 0.001. n.s. = not significant. Copyright 2018 [46], the American Chemical Society. (**C**) DNA hydrogel incorporated with gold nanorod for cancer drug delivery. Reproduced with permission. Copyright 2015 [48], Royal Society of Chemistry. (**D**) Schematic illustration of RNA interference (RNAi) caused by RNAi-exhibiting gel (I-gel) in living cells. Reproduced with permission. Copyright 2018 [47], Springer Nature.

A variety of therapeutic genes and cytosine-phosphate-guanine (CpG) motifs can be incorporated into branched DNA monomers. As a result, the prepared DNA hydrogels can be applied in gene therapy and immunotherapy of cancer. To construct targeted gene delivery vectors, Li and coworkers designed two Y-monomers, DNA linkers and integrated aptamers, disulfide bonds, and therapeutic genes into Y-monomers, so that the prepared DNA hydrogels were capable of delivering therapeutic genes to specific target cells for the treatment of cancer [45]. The self-assembled DNA hydrogels could target specific tumor cells via aptamers recognition. Furthermore, the disulfide bond endowed the DNA hydrogels with special stimulus responsiveness. As a reducing agent in the cytoplasm, reduced glutathione (GSH) could degrade the hydrogels after they are taken up by tumor cells, thus achieving the selective release of antisense oligonucleotides (ASOs), ribozymes and other therapeutic genes in the cells. Shao and coworkers developed an injectable DNA supramolecular hydrogel vaccine (DSHV) system formed from Y-scaffolds and DNA linkers [46]. CpG motifs served as a linker and was incorporated into Y-scaffolds DNA, the hydrogel constructed with these monomers could effectively recruit and activate antigen presenting cells (APC) to produce a variety of cytokines in vitro and in vivo (Figure 2A). Experiments showed that the DSHV system could stimulated APCs to secrete 365 pg/mL Interleukin (IL)-6 and 12 pg/mL IL-12, which was comparable to that in 5 μg/mL lipopolysaccharide treated groups. Therefore, DSHV showed a remarkable efficacy in tumor immunotherapy through enhancing immune response (Figure 2B).

Therapeutic agents and nanoparticles with antitumor effects can also be incorporated into branched DNA hydrogels via chemical crosslinking or physical interaction. These drug delivery systems possess the capability to achieve selective drug release in vivo. Zhang et al. coupled camptothecin, a natural anticancer drug, to the backbones of phosphorothioate DNA [66]. These DNA strands could self-assemble into two types of Y-shaped building blocks, which were then cross-linked to form injectable drug-containing hydrogels. In addition to chemical crosslinking, electrostatic interaction was usually used as a material loading method as well. In the study conducted by Park group, they utilized X-shaped DNA to form a highly negatively charged nanogel, which could effectively load positively charged gold nanorods (AuNRs) through electrostatic attraction, resulting in a stable AuNRs-loaded DNA hydrogel [48]. The small molecule drug DOX was subsequently loaded into the material. The gels could release AuNRs and DOX through near-infrared response, which was an anti-tumor strategy combined with chemotherapy and photothermal therapy (PTT) (Figure 2C). The relative tumor volume of mice treated with DOX-AuNRs-DNA hydrogels was 3.9 times that of the PBS group, showing that DOX-AuNRs-DNA hydrogels could effectively inhibit the tumor growth. Besides traditional anticancer drugs, glucose oxidase (GOx) also could be loaded in the branched DNA hydrogel, which could deplete glucose and trigger starvation therapy and provide more diversified strategies for cancer therapy [67].

Different therapeutic drugs can be co-loaded in DNA hydrogels to achieve combined therapy of tumors. Wei et al. designed cruciform DNA (C-DNA) containing a pH-responsive i-motif structure and a fusion sequence including MUC1 aptamer and CpG oligodeoxynucleotides (ONDs) [68]. DOX could be inserted into assembled DNA nanogels, resulting in hybrid materials with precise delivery, powerful immunostimulatory activity, and chemotherapeutic effects. The DNA tetrahedral nanogels prepared by Tang et al. were assembled from aptamer-functionalized DNA tetrahedrons as monomers and ASOs as crosslinking agent [69]. Then DOX was loaded into the hydrogel. DOX and ASOs could release through the reduction response of GSH to disulfide bonds, which could inhibit multidrug resistant tumors.

In addition to delivering small molecule drugs, branched DNA hydrogels can also be used to build systems which could produce RNA and proteins [27], thus providing a platform for the efficient production of anti-tumor nucleic acids and proteins in cells. Song et al. integrated plasmid DNA in branched X-shaped DNA (X-DNA) formed hydrogel (I-gels). The plasmids in I-gels could transcribe siRNA, to evaluate the RNA interference (RNAi) efficiency of I-gels, they examined the green fluorescent protein (GFP) interference effect. The results showed that I-gels possessed 9.4 times and 2.8 times higher RNAi efficiency than free plasmids and plasmids-complexed liposomes, respectively (Figure 2D) [47].

### 2.2. HCR-Synthesized DNA Networks Formed Hydrogel

#### 2.2.1. HCR-Synthesized DNA Networks

**Figure 3:**
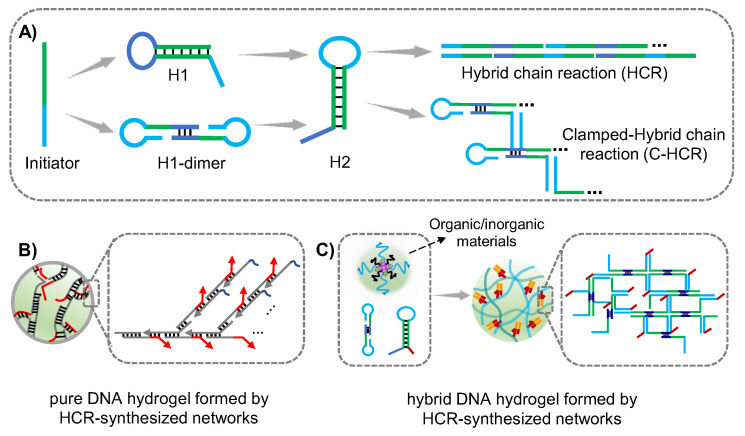
(**A**) The schematic of HCR principle. (**B**) Pure DNA hydrogel formed by HCR-synthesized networks. (**C**) Hybrid DNA hydrogel formed by HCR-synthesized networks.

HCR is one of isothermal amplification methods without enzyme participation, and was proposed by Pierce and Dirks in 2004 [70]. The HCR would spontaneously proceed due to the Gibbs free energy driving force. In the HCR system, hairpins H1 and H2 undergo an alternating reaction of strand substitution in the presence of a trigger strand initiator, forming a long double-stranded DNA structure based on the principle of base pairing. At present, a variety of signal amplification technology based on HCR have been developed. Wang group prepared supramolecular DNA scaffolds on the surface of cell membranes via HCR strategy for the protection of mammalian cells, and ultra-sensitive detection methods of extracellular vehicles (EVs) were developed based on aptamer targeting combined with HCR amplification technology [71,72]. By designing complementary sequences between DNA linkers, long DNA strands obtained from HCR can be cross-linked to form DNA hydrogels, however, the crosslinking efficiency could be affected by the complex conformation and steric hindrance due to the functional motifs which could only be designed at the end of hairpins. Generally, the initiator strand is designed to anchor on the interface of carriers such as bio-interface and gold nanoparticle (AuNPs) interface. When hairpins were introduced, the HCR would be triggered and DNA hydrogel could be formed. In addition, the initiator could be modified and hybridized with other organic/inorganic polymers to prepare hybrid HCR-synthesized DNA networks-based hydrogel (Figure 3).

#### 2.2.2. Application in Cancer Therapy

**Figure 4:**
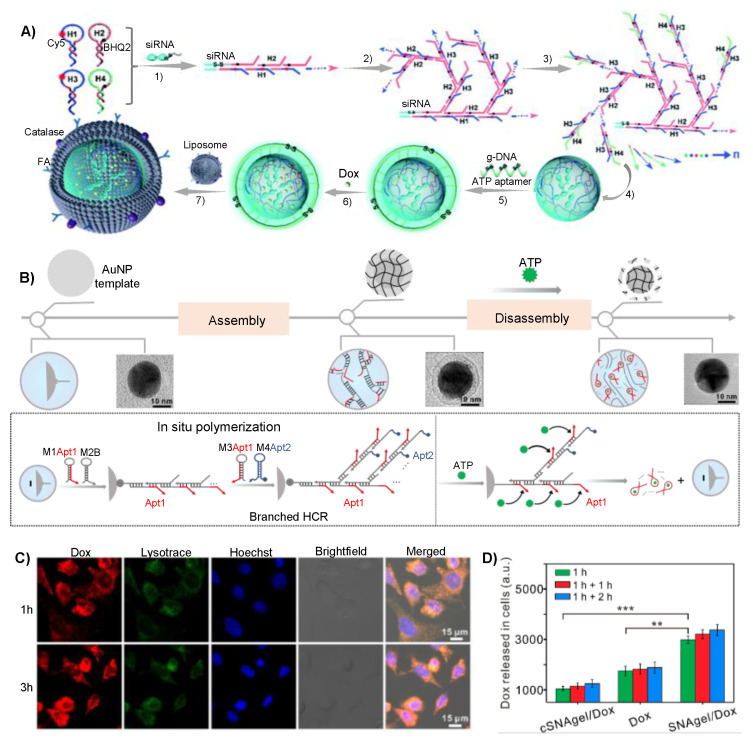
Pure DNA hydrogel prepared through HCR strategy for cancer therapy. (**A**) Schematic of the design and preparation of core-shell spherical 3D DNA hydrogel via HCR for synergistic cancer therapy. Reproduced with permission. Copyright 2021 [49], the American Chemical Society. (**B**) Spherical nucleic acid-templated hydrogel (SNAgel) via HCR strategy for programming controllable DOX delivery. (**C**,**D**) ATP triggered intracellular burst release of SNAgel. Reproduced with permission. ** *p* < 0.01, *** *p* < 0.001. Copyright 2019 [50], the American Chemical Society.

As mentioned before, the key issue of a drug delivery system is to establish biocompatible and release-controllable drug carriers. Based on HCR amplification technology, Na group prepared a core-shell spherical 3D DNA hydrogel for synergistic cancer therapy [49]. The siRNA was used as initiator strand to prepare DNA cores, then hairpins H1, H2, H3, and H4 were added for polymerization (Figure 4A). The DNA hydrogel core was encapsulated by a liposome membrane functionalized with catalase and folic acid, enabling synergistic targeting of catalase and functional folic acid chemotaxis. Compared with traditional passive or active targeting, the synergistic targeting greatly enhanced the cellular uptake of targeted cells. Furthermore, ATP and GSH responsiveness were achieved by ATP aptamers and S-S bonds, respectively. H1 and H3 hybridized with Survivin mRNA to release siRNA and DOX for synergistic cancer therapy. Pei and coworkers developed a switch-engineered spherical nucleic acid-templated hydrogel (SNAgel) that achieved precise control of drug release through dissociation of the DNA shell by ATP triggered structural transformation of DNA switch (Figure 4B–D) [50]. The DOX could be rapidly released due to the ATP-triggered conformational change and consequent the dissociation of SNAgel. In the presence of ATP, about 80% of the loaded DOX was released from SNAgel. In addition, the author found that the kinetic control of drug release could be achieved by adjusting the length of toehold sequences. The novel controlled burst release strategy reduced the drug dose and increased drug efficacy, demonstrating great potential for precise cancer therapy.

**Figure 5:**
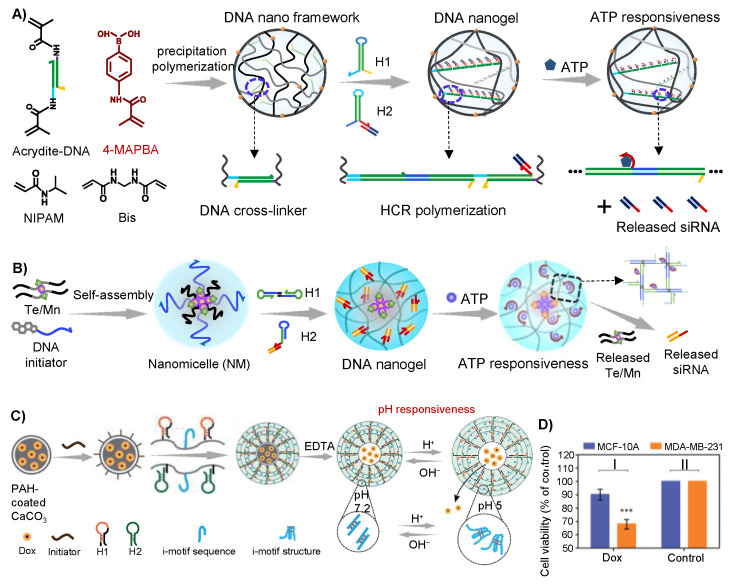
Hybrid DNA hydrogel prepared through HCR strategy for cancer therapy. (**A**) Schematic of the preparation and drug release of hybrid DNA nanogel. Reproduced with permission. Copyright 2021 [52], Nature Portfolio. (**B**) The principle of hybrid DNA nanogel based on C-HCR-synthesized networks. Reproduced with permission. Copyright 2021 [51], Wiley-VCH Verlag GmbH. (**C**) The schematic of pH-responsive hybrid DNA hydrogel for the DOX release. (**D**) Cytotoxicity assay of the pH-responsive hydrogel loaded with DOX into MCF-10A cells and MDA-MB-231 malignant breast cancer cells. *** *p* < 0.001. Reproduced with permission. Copyright 2017 [53], Royal Society Chemistry.

Hybrid DNA hydrogel could also be prepared via the hybridization between DNA molecules and other polymers. We recently developed a hybrid DNA nanogel for siRNA delivery via HCR. In our work, we firstly prepared DNA cross-linked polymeric nano-framework (DPNF) through precipitation polymerization method. When the designer harpins DNA H1 and H2 were introduced into the solution, cascade hybridization between harpins DNA H1 and H2 could be initiated by the DNA cross-linkers in the DPNF and DNA nanogel could be formed (Figure 5A). The harpin H2 was designed to tether with siRNA sequences, thus resulting successful loading of siRNA in DNA nanogel along with the cascade assembly of H1 and H2. The siRNA loaded DNA nanogel could be easily uptaken by tumor cells and escaped from lysosome to cytoplasm with the mediating of methacrylamidophenyl (MAPBA) on the polymer framework. Moreover, the tethered sequences between siRNA and H2 were designed as ATP aptamer, and when the DNA nanogel-siRNA reached cytoplasm, the release of siRNA could be specially triggered via ATP. Moreover, both the in vitro and in vivo experiment showed the significant gene knockdown of PLK1 which was overexpressed in tumor cells [52]. Nanomicelles are colloidal constructures composed of amphiphilic monomers that usually have a small hydrophobic head and a long hydrophilic tail [73]. The nanomicelles could also encapsulate hydrophobic drugs to improve the imaging and diagnostic sensitivity. We discovered that DNA hybrid micelle nanogel could be successfully achieved by using cascade clamped hybridization chain reaction (C-HCR) technology, thus effectively realizing the loading and delivery of siRNA. The core of the nanomicelle was co-assembled with tellurium/Mn(II) containing amphipathic molecules and cholesterol-modified DNA, and the DNA was exposed on the outside of the micelle. In the presence of the micelle, the designer hairpins H1 and H2 would be initiated to assemble inside the micelle via C-HCR strategy, in which process the siRNA tethered on H2 could be assembled in the formed DNA network on the outside of the micelles (Figure 5B). The tellurium/Mn(II) containing amphipathic molecules in the core could catalyze Fenton-like reaction of hydrogen peroxide to produce hydroxyl radicals, which could combine with the siRNA generated gene regulation to inhibit the progression of cancer. The DNA hybrid nanogel achieved synergistic chemical and gene regulation based on C-HCR process, which provided a novel strategy for cancer therapy and illustrated that the C-HCR strategy could apply to drug delivery systems [51]. In addition, Willner and his colleagues prepared a pH-responsive DNA-acrylamide hybrid hydrogel microcapsules via assembly of DNA on poly(allylamine hydrochloride) (PAH)-CaCO_3_ nanoparticles, and thereby achieved efficient loading and controlled release of the chemotherapeutic drug DOX [53]. The initiating DNA chain was adsorbed on the DOX-loaded PAH-CaCO_3_, and when hairpins H1 and H2 were introduced, the HCR reaction was then triggered on the surface of PAH-coated CaCO_3_ to form a coating layer. Then the PAH-CaCO_3_ core was dissolved by adding ethylenediaminetetraacetic acid (EDTA) to form DOX-loaded DNA microcapsule drug delivery system. The formation and dissolution of this microcapsule could be achieved through pH-responsiveness based on the formation of i-motif structure under acidic conditions, which would reduce the stiffness of the microcapsule hydrogel layer to enhance its fluidity, thus realizing the release of DOX (Figure 5C). The cytotoxicity assay demonstrated high cytotoxicity of pH-responsive hydrogel microcapsule towards MDA-MB-231 cells, which exhibited ca.35% death rate after a five-day interval (Figure 5D). The strategy demonstrated the good biocompatibility and efficient drug loading and delivery capability of the DNA hydrogel-based drug delivery system, which presented the unique advantages of stimuli-responsive DNA structures in certain conditions.

### 2.3. RCA-Produced DNA Chain-Based Hydrogel

#### 2.3.1. RCA-Produced DNA Chain

**Figure 6:**
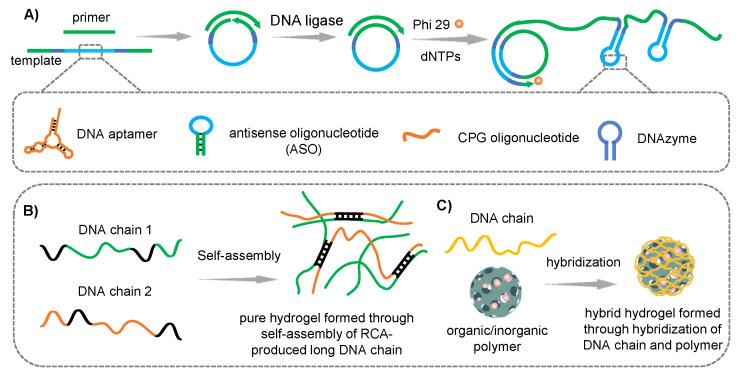
(**A**) The schematic of RCA principle. (**B**) Pure hydrogel formed through self-assembly of RCA-produced long DNA chain. (**C**) Hybrid hydrogel formed through hybridization of DNA chain and organic/inorganic polymer.

Besides branched DNA modules assembly and HCR-synthesized DNA networks crosslinking, another universal assembly strategy for the preparation of DNA hydrogel is rolling circle amplification (RCA). RCA is an isothermal enzyme-based amplification method to synthesize ultralong DNA strands, with template strand, primer, and phi29 enzyme as the main components. The entire amplification process involves three steps: annealing to form circle DNA template, T4 ligase connection and phi29 enzyme-based amplification. RCA amplification method has been extensively used in cell engineering fields such as the capture of cells, detection of miRNA and the delivery of small molecules [74]. Zhang et al. used artificial DNA base to fabricate DNA nanoflowers (DNFs) using RCA technology, they incorporated a ferrocene base inside the DNFs to manipulate the size of DNFs and endowed DNFs with self-degradability via Fenton’s reaction, thus achieving the improvement of the therapeutic efficacy [75]. Through physically intertwining of two complementary long DNA strands, or hybridization of RCA product with other organic/inorganic materials, RCA chain-based DNA hydrogel could be formed (Figure 6). This DNA hydrogel is easy to synthesize because of the simple reaction process, wherein the template and primer sequences require rational and accurate design.

#### 2.3.2. Application in Cancer Therapy

Benefitting from the designable property of RCA template sequence, different therapeutic agents could be loaded and delivered. In addition to chemotherapeutic drugs and small molecule nucleic acid drugs, functional sequences such as CPG and ASO sequences could be designed and amplified via RCA as well, providing more treatment options for cancer therapy. Here in this section, RCA-produced long DNA chain-based DNA hydrogel for chemotherapy, gene therapy, immunotherapy, photo dynamic/thermal therapy, and cooperative therapy are summarized.

**Figure 7:**
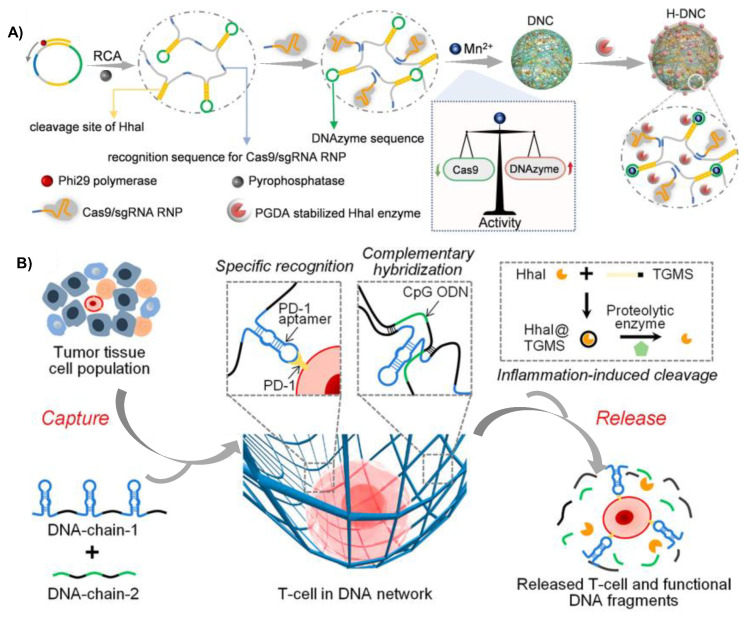
RCA technology-based DNA hydrogel for cancer therapy. (**A**) The principle of RCA strategy-based DNA nanogel for the co-delivery of DNAzyme and CRISPR/Cas 9 for gene therapy. Reproduced with permission. Copyright 2022 [55], Wiley-VCH Verlag GmbH. (**B**) Double RCA assembly strategy formed DNA hydrogel for the isolation and release of T cells for cancer immunotherapy. Copyright 2021 [56], the American Chemical Society.

Gu and coworkers prepared a biodegradable DNA nanogel based on RCA technology for the tumor chemotherapy, the DNA nanogel consisted of DNA nanoclew (NCl) formed from long single RCA strand and acid-responsive nanocapsules (Nca) containing DNase 1 [54]. NCl was designed with more G-C base pairs for enhancing the loading capacity of DOX. The author demonstrated the endocytosis pathway of the DNA nanogel and when the DNA nanogel was taken up by cancer cells MCF-7 through folic acid (FA) mediated endocytosis, the Nca polymer shell would be degraded under the lysosome acidic microenvironment of lysosomes. Then, the released Dnase 1 would accelerate the degradation of NCl, resulting in the quick release of the encapsulated DOX within 30 min, thus enhancing the release efficiency. Gene therapy has attracted much attention since the burst development of drug delivery systems and nucleic acid drugs [76]. We recently developed a hybrid DNA nanogel which enabled co-delivery of DNAzyme and CRISPR/Cas9 for the combined gene therapy of breast cancer [55]. Repeated DNAzyme sequences, sgRNA recognition sequences, and the cleavage sites for Hhal enzyme that could cleave miRNA inside cancer cells were obtained via specifically designing the template sequence of RCA. Mn^2+^ was added as the cofactor of DNAzyme to compress the long DNA chain into nanoparticles, and DNA nanogel was then formed by mixing nanoparticles with Hhal enzyme coated acid-responsive polymer (Figure 7A). After being taken up by cancer cells, the acidic environment within lysosomes would trigger the degradation of the polymer coating, subsequently exposing the Hhal enzyme, which would recognize and cleave the specific sites, thereby releasing the Cas9/sgRNA and DNAzyme for combined gene therapy. Based on RCA and rolling circle transcription (RCT) technologies, Lee et al. prepared a DNA-RNA hybrid hydrogel through stepwise double enzymatic polymerization [77]. The DNA-RNA hybrid hydrogel exhibited ultra-soft mechanical properties with an elastic modulus of approximately 100 Pa, indicating that the hydrogel was easy to inject. In physiological condition, the hydrogel network could be cleaved by restriction enzymes to release the siRNA-AS1411 aptamer complex polymer, enabling gene therapy. Immune checkpoint inhibitors such as anti-programmed cell death protein 1 (PD-1)/PDL-1, and CPG oligonucleotides are commonly used in immunotherapy. The critical aspect of immunotherapy is the efficient capture and isolation of immune cells with minimal damage and high purity [78]. Gu group developed a DNA nano-cocoon hydrogel based on RCA strategy for the combination delivery of PD-1 antibody and CPG oligonucleotides. The DNA nano-cocoon achieved controlled release of PD-1 antibody and CPG oligonucleotides, which appeared considerable immune response and therapy efficiency [79]. RCA technology-based DNA hydrogel provided a physiological environment and could be designed to generate repeated PD-1 aptamer and CPG oligonucleotides, which was a more cost-effective alternative to antibodies. In 2021, Yang group developed a DNA hydrogel network for the specific cell isolation and in situ cell culture of T lymphocytes (T cells) on the basis of RCA principle (Figure 7B) [56]. DNA hydrogel network was formed by two cross-linked ultralong DNA strands with complementary sequences (Strand 1 and Strand 2). Strand 1 was designed to contain the sequence of PD-1 aptamer, and strand 2 contained the CPG oligonucleotides sequences. The cross-linked DNA hydrogel network could specifically recognize the receptor on the surface of T cells and encapsulate them, while also providing cleavage sites for restriction enzyme to facilitate the responsive release of T cells in cancer immunotherapy. The purity of T cells isolated by this strategy was up to 98% and the viability of released T cells maintained above 90%, which was expected to be used in clinical study and treatment.

**Figure 8:**
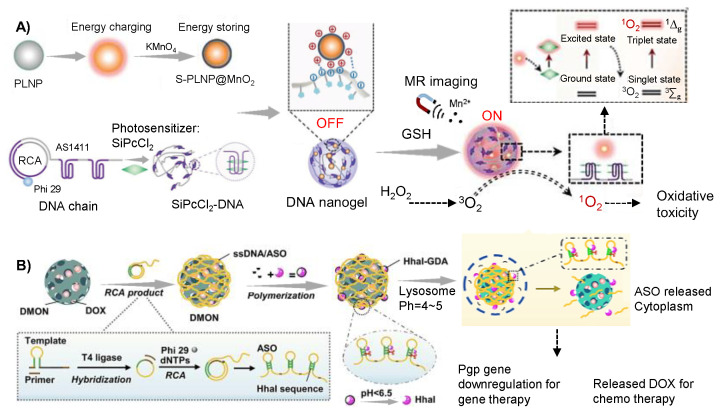
RCA technology-based hybrid DNA hydrogel for cancer therapy. (**A**) DNA hybrid nanogel for the PDT of breast cancer. Reproduced with permission. Copyright 2022, Wiley-VCH Verlag GmbH [57]. (**B**) The schematic of DNA hybrid nanogel (DMON/DOX-DNA/ASO-HhaI@GDA) for the chemo and gene cooperative therapy. Reproduced with permission. Copyright 2022 [58], the American Chemical Society.

The advancement of DNA nanotechnology has propelled the progress of photodynamic therapy (PDT) and PTT. Conventional PDT techniques require external light irradiation, however, the low tissue penetration ability of light has limited its use in cancer treatment [80]. Yang and coworkers recently developed an exogenous laser excitation independent DNA hybrid nanogel for the PDT of breast cancer [57]. Based on RCA technology, AS1411 aptamers targeting the receptors on cancer cell surface were amplified and loaded with the photosensitizer SiPcCl_2_. To achieve PDT without the need for an external light source, persistent-luminescence nanoparticles (PLNPs) coated with MnO_2_ were designed as self-illuminants, capable of storing energy upon energy supplementation. When the GSH overexpressed in cancer cells respond to the MnO_2_ coating on PLNPs surface, MnO_2_ would be restored to O_2_, allowing PLNPs to activate SiPcCl_2_ and convert O_2_ to cytotoxic ^1^O_2_, thus achieving effective PDT therapy (Figure 8A). Results showed that the tumor suppression rate of the SiPcCl_2_-loaded DNA nanohydrogel was more than 80%. Apart from PDT, they also developed DNA-polydopamine-MnO_2_ hybrid nanogel for the cancer gene therapy and PTT [81]. DNA hybrid nanogels were composed of DNA nanoflower containing DNAzyme sequences that obtained from RCA and polydopamine-MnO_2_ (PM) complex. PM would induce an increase in temperature in tumor site through photothermal conversion under near-infrared-light radiation and achieve PCT. Simultaneously, GSH in tumor cells would reduce MnO_2_ into Mn^2+^, thus activating the DNAzyme and enhancing the cleavage activity of DNAzyme on Egr-1 mRNA, which would down-regulate Egr-1 protein in tumor cells and achieve gene therapy. The gene-photothermal synergistic therapy strategy facilitated the development of DNAzyme-based gene therapy. Yao et al. recently developed a DNA hybrid nanogel (DMON/DOX-DNA/ASO-HhaI@GDA) for the chemo and gene cooperative therapy [58]. The DOX/gene delivery system consisted of an acid-responsive Hhal degradable hydrogel layer (Hhal@GDA) and a GSH-sensitive dendritic mesoporous organosilica nanoparticle (DMON). Hhal@GDA contained repeated ASO sequences formed via RCA, while DMON was used for DOX loading (Figure 8B). Upon endocytosis of the DOX-loaded hybrid nanogel into tumor cells, the Hhal@GDA layer would degrade in the acidic lysosomal environment, which would active Hhal to cleave the ultralong DNA chain and release the gene drugs ASOs, thus down-regulating the P glycoprotein expression. Simultaneously, S-S bonds contained in DMONs would respond to the intracellular GSH to trigger the release of chemotherapy drugs DOX, thus enabling a synergistic drug delivery approach and facilitating cooperative cancer therapy.

## 3. Conclusions and Perspectives

In this review, we summarized the construction strategies of DNA hydrogels on the basis of branched DNA modules, HCR-synthesized DNA networks and RCA-produced DNA chains, respectively. Benefiting from the programmability of DNA sequence, the synthesized DNA hydrogels not only exhibit well mechanical property and adjustable size tunability, but could achieve specific recognition capacity and good biocompatibility as well. These properties endow DNA hydrogels with drug loading capability and controlled release ability, which make DNA hydrogels as superior candidate of drug delivery carriers for cancer therapy. With rational design, DNA hydrogels can be easily functionalized and endowed with fascinating multiple responsiveness, which contribute to the selective release of drugs in specific environments. Apart from traditional chemotherapeutics, small nucleic acid molecules with cancer therapy effects could also be designed and encapsulated in DNA hydrogels; upon specific trigger, the loaded drugs could be released and specific cancer therapy such as chemotherapy, gene therapy, immune therapy, photo dynamic/thermal therapy, and cooperative cancer therapy could be achieved.

Despite the notable progress of DNA hydrogels in cancer therapy, there are still several limitations that hinder their clinical transformation. Firstly, in order to realize the clinical applications of DNA hydrogels in drug delivery, the cost of producing DNA should be considered. Currently, it is challenging to synthesize DNA hydrogels in large quantities at a low cost. Thus, more systematic and efficient synthetic strategies should be developed to build high-quality, high-throughput, and low-cost DNA production platforms. Secondly, it is crucial to conduct comprehensive research on the biological stability, pharmacology, toxicity, and animal pharmacokinetics of DNA hydrogels to assess their clinical application. Nevertheless, the pharmacokinetic studies of DNA hydrogels was insufficient. The distribution, metabolism, and degradation of DNA hydrogels in vivo require further investigation, furthermore, the efficacy and safety of DNA hydrogels in the clinical applications need to be thoroughly evaluated. Finally, the release kinetics of drugs from DNA hydrogels, which can guide the controlled delivery of drugs, has rarely been reported, and precise kinetic research is needed to provide theoretical support for the development of responsive hydrogels with controlled release properties. In summary, as a biomacromolecule originating from organisms, DNA can serve as an adjustable bridge between macro and micro scale structures through specific and rational molecule design. DNA hydrogels obtained from DNA or DNA-involved hybrid structures have shown broad prospects in targeted drug delivery for cancer therapy. We anticipate that DNA hydrogels will continue to drive the development of intelligent therapeutic systems due to their unique advantages.

## Figures and Tables

**Table 1:** The summary of DNA hydrogels formed via branched DNA modules, hybrid chain reaction (HCR)-synthesized DNA networks and rolling circle amplification (RCA)-produced DNA chains, respectively, and their application of drug delivery for cancer therapy.

Type of DNA Hydrogels	Formulation	Strategy Advantages	Strategy Limitations	Delivered Drugs	Application in Cancer Therapy	Ref.
Non-enzyme-mediated branched hydrogel	stimuli responsiveness (pH, temperature)	controllable symmetry; multivalency; enzyme-free	high concentration for preparing hydrogel	ASOs; CPG	immunotherapy	[45,46]
Enzyme-mediated branched hydrogel	strand extension (ligase)	controllable symmetry; multivalency; short reaction time	high concentration for preparing hydrogel; high cost	Camptothecin; DOX; transcribed siRNA	chemotherapy; chemo-photo thermal synergistic therapy gene therapy	[47,48]
Pure hydrogel formed by HCR-synthesized networks	linear/clamped amplification	isothermal amplification; enzyme-free; convenient operation	high requirements for sequence design; require reaction carrier (AuNP, cell membrane, …) for HCR	DOX; siRNA	chemo-gene synergistic therapy; chemotherapy	[49,50]
Hybrid hydrogel formed by HCR-synthesized networks	hybrid with other organic/inorganic material before HCR amplification	isothermal amplification; enzyme-free; high stability	high requirements for sequence design; lower initiation efficiency influenced by complex conformation	siRNA; DOX	gene therapy; chemotherapy	[51,52,53]
Pure hydrogel formed by RCA-produced long DNA chain	physical crosslinking after RCA reaction	isothermal amplification; convenient operation	high requirements for sequence (template and primer) design; high cost low stability	DOX; DNAzyme and CRISPR/Cas9 system; siRNA; CPG	chemotherapy; gene therapy; immunotherapy	[54,55,56]
Hybrid hydrogel formed by RCA-produced long DNA chain	hybrid with other organic/inorganic material after RCA amplification	isothermal amplification high stability	high requirements for sequence (template and primer) design high cost; complex operation for hybridization	SiPcCl_2;_ DOX; ASOs	gene-photo thermal synergistic therapy; chemo-gene synergistic therapy	[57,58]

## Data Availability

The data presented in this study are available on request from the corresponding author.
